# Examination of exhaustive cloning attempts reveals that *C. elegans* piRNAs, transposons, and repeat sequences are efficiently cloned in yeast, but not in bacteria

**DOI:** 10.3389/fgene.2014.00275

**Published:** 2014-08-28

**Authors:** Or Sagy, Ron Shamir, Oded Rechavi

**Affiliations:** ^1^Department of Neurobiology, Wise Faculty of Life Sciences and Sagol School of Neuroscience, Tel Aviv UniversityTel Aviv, Israel; ^2^Blavatnik School of Computer Science, Tel Aviv UniversityTel Aviv, Israel

**Keywords:** *C. elegans*, bacteria, lateral gene transfer, piRNAs, PIWI-interacting small RNAs, repetitive elements

## Abstract

Genome sequencing requires insertion of random fragments of the sequenced organism’s DNA into a unicellular host, most often *Escherichia coli* bacteria. This manipulation was found in the past to be analogous to naturally occurring horizontal gene transfer, and moreover has proved valuable to understanding toxicity of foreign genetic elements to *E. coli*. Sequencing of the *Caenorhabditis elegans* genome was similarly achieved via DNA transformation into *E. coli*. However, numerous attempts have proven a significant percentage of the genome unclonable using bacteria, although clonable via yeast. We examined the genomic segments that were not clonable in bacteria but were clonable in yeast, and observed that, in line with previous hypotheses, such sequences are more repetitive on average compared with the entire *C. elegans* genome. In addition, we found that these gap-sequences encode significantly more for DNA transposons. Surprisingly, we discovered that although the vast majority of the *C. elegans* genome is clonable in bacteria (77.5%), almost all the thousands of sequences that encode for PIWI-interacting small RNAs, or 21U-RNAs (91.6%) were only clonable in yeast. These results might help understanding why most piRNAs in *C. elegans* are physically clustered on particular loci on chromosome IV. In worms and in a large number of other organisms, piRNAs serve to distinguish “Self” from “Non-Self” sequences, and thus to protect the integrity of the genome against foreign genetic elements, such as transposons. We discuss the possible implications of these discoveries.

## INTRODUCTION

During the 1990s and early 2000s, there was a race to sequence the human genome (Collins et al., 2003). The required techniques were developed in a rapid pace, and in parallel utilized for sequencing of other multicellular organism’s genomes. The genome of the nematode *Caenorhabditis elegans* (*C. elegans*) was the first one completed (The *C. elegans* Sequencing Consortium, 1998). An unexpected result of the development of the different DNA cloning techniques was the accumulation of negative results, failed cloning attempts that allow gaining insight regarding barriers for genomic information transfer between organisms.

As part of sequencing the *C. elegans* genome, the whole genome of the worm was randomly broken into overlapping fragments, which were transformed into *Escherichia coli* bacteria through the use of very large cloning vectors termed cosmids and fosmids (Coulson et al., 1986; Kim et al., 1992; Perkins et al., 2005). With sufficiently high coverage, sequencing of multiple overlapping fragments should allow in theory the assembly of the corresponding full genome. During the project, researchers soon realized that certain parts of the worm’s genome could not be cloned in the bacteria, leaving gaps in the resultant genome (Coulson et al., 1991). Even though there have been numerous attempts over the past 20 years at filling the gaps using cosmids and fosmids [including a consortium dedicated to creating a library of fosmids that would cover the entire genome (Perkins et al., 2005)], none have been successful in covering these gaps. In total, about 20% of the genome could not be cloned in bacteria in spite of these repeated efforts. Throughout this manuscript we will refer to such sequences as “gap sequences.”

In the 1990s, in order to close the gaps and obtain a complete sequence of the *C. elegans* genome, researchers turned to cloning in yeast, by using YACs [Yeast Artificial Chromosomes (Coulson et al., 1991)]. The gap sequences quickly proved clonable in yeast (*Saccharomyces cerevisiae*), and thus YACs that contain sequences covering the gaps were rapidly obtained and facilitated the completion of the worm genome (Wilson, 1999). Even so, the reason for the “non-clonability” of these gaps in bacteria largely remained a mystery.

We found this incompatibility of worm genome fragments with bacterial cloning a valuable resource. *C. elegans* nematodes feed on bacteria both in real-life and in lab conditions, and this close contact facilitates exchange of genetic information between the nematodes and their resident microbes. For example, bacterially-expressed double-stranded RNA transfers from the bacteria to the worm and silences endogenous *C. elegans* genes (Timmons et al., 2001; Mello and Conte, 2004; Liu et al., 2012; Sarkies and Miska, 2013). Although lateral gene transfer has been most extensively described in microorganisms, recent studies suggest that the process occurs more frequently than previously appreciated in eukaryotes as well (Bruto et al., 2014). DNA sequences were also shown to be laterally exchanged between nematodes and symbionts (Haegeman et al., 2011). This is consistent with the various findings of gene exchange between organisms at all evolutionary distances (Boto, 2010; Koonin, 2010). Thus, a side benefit of the artificial cloning procedure used for sequencing the *C. elegans* genome is that it provides a keyhole into limitations of natural genome transfer between worm and bacteria.

Given that the aforementioned gaps have not been clonable in *E. coli*, a prokaryote, but have been clonable in the eukaryote *S. cerevisiae*, we hypothesized that there would be a genetic factor in these regions causing the adopting prokaryotic host to die. This notion was inspired by Sorek et al’s. (2007) experimental research on inhibition of gene transfer between different bacterial genomes to *E. coli*. Such analysis of clone insertions and cloning gaps has proved valuable to understanding toxicity of bacterially derived genetic elements to *E. coli* in the past (Sberro et al., 2013).

It has been believed that the cause of the lack of clonability of various regions of the *C. elegans* genome was their high degree of repetitiveness (Wilson, 1999). We set out to explore whether there was another underlying property of these regions that, possibly in conjunction with repetitiveness, was actively inhibiting the prokaryotic hosts of these genome sequences from thriving.

## MATERIALS AND METHODS

### PRECISE ALIGNMENT OF THE CLONES TO THE GENOME

The sequences of *C. elegans* clones were downloaded from NCBI BioProject PRJNA13758, and the precise location of each of the clones was determined by BLASTing the sequence to the *C. elegans* genome (version WBcel235; this step was required as the locations in the NCBI record did not fit the genome version that we utilized). We used clone sequences from WormBase, retrieved from NCBI on April 4th, 2013. The sequences were categorized as “Cosmid,” “Fosmid,” or “YAC” clones.

The YAC subsequences that we analyzed tended to bridge across gap sequences, with both YAC ends occasionally overlapping with finished cosmid/fosmid sequences. The overlap varied between 0% and 100% (3 YAC sequences were found to be covered fully by cosmids/fosmids – Y119C1C, Y110A2B, Y70C5B). On average ∼7% of the YAC sequence’s ends overlapped with cosmids/fosmids. To prevent this overlap from influencing our results, we removed the overlapping segments from the YAC sequences before running any analyses. The resulting “YAC-exclusive” sequences were used in our study (hereby referred to as “YACs”).

### REPEATMASKER ANALYSIS

As a first step, and in accordance with previous notions of repetitiveness as the factor limiting cloning of the YAC sequences, we ran RepeatMasker separately on YACs, cosmids, and fosmids. This was conducted for each of the individual clones. We compared the average, median, minimal, maximal, and non-zero results of each RepeatMasker parameter for every clone type (RepeatMasker was run with the species set as “*C. elegans”*). RepeatMasker lists retroelements (including sub-classifications), DNA transposons (including sub-classifications), rolling-circles, other unclassified interspersed repeats, small RNA, satellites, simple repeats, and low complexity regions (Smit et al., 1996–2010). As a control test for each clone type we also generated a random library with the same number of clones taken from random segments of the *C. elegans* genome, (hereby ”random clones”), of similar sizes as those comprising that clone type (based on the average length and length standard deviation). These libraries were examined using the same RepeatMasker analysis. This analysis was used to assess the significance of our results, in comparison to enrichments detected by chance.

### COMPARISON BY GENOMIC LOCATION

It has been observed that the distribution of repeat elements along chromosomes is highly uneven (The *C. elegans* Sequencing Consortium, 1998). We therefore evaluated the difference in repeat element distributions between YACs and cosmids, by taking into account the location of the clones. The purpose of this analysis was to reduce bias caused by different location distributions of the types of clones. It may be hypothesized that these differences were causing the disparity that we found. In this, and the next couple of analyses, we compared YACs to cosmids and ignored fosmids, as there were much fewer fosmids compared to cosmids (107 vs. 2,520). To measure the effect of the clone’s location along the chromosome and to obtain robust statistical analysis, we partitioned each chromosome into four equal-size quarters and summarized the statistics of the two extreme quarters together and of the two middle ones together.

### COMPARISON BY CLONE LENGTH

Another factor that we took into consideration was the clone’s length. Although YACs are generally much longer than cosmids, the YAC segments that were evaluated here were shorter, as they were trimmed (as mentioned above) to contain only the gaps used to bridge between cosmids. The YAC-exclusive segment median length was roughly 23.7 kb, while for the cosmids the median length was approximately 32.5 kb. In this analysis we divided each clone type into two groups – one of clones below the median length of the clone type and the other of clones over the median length. Statistics were collected for each group.

### COMPARISON OF GENES ENCODED IN THE CLONES

We categorized the genes in the clones based on the type of product that they encode for (or do not encode for): coding sequences (CDS), non coding RNAs (ncRNA), tRNAs, and rRNAs. This categorization was determined based on the NCBI classification of each gene.

## RESULTS

By comparing the sequences that were cloned in YACs, cosmids and fosmids, we observed, consistently with previous reports, that YAC sequences were overall more than twice as repetitive as the sequences cloned in cosmids. On average, 21.42% of the YAC nucleotides vs. 10.15% of cosmid nucleotides were masked by RepeatMasker. The most abundant repeat category, and an interesting result by itself, was enrichment in DNA transposons of various types in the YAC sequences (See **Table 1**). On average, 11.44% of YAC sequences were found to be DNA transposons vs. 5.69% in cosmids. Of the transposons, the most major difference was in PiggyBac transposons – 1.53% in YACs on average vs. 0.48% in cosmids. Other than the transposons, significant differences were found in unclassified repeats (4.65% vs. 1.58%) and satellites (2.52% vs. 0.51%). The content of Simple repeats also varied – 1.65% vs. 1.06%. The total interspersed repeats (all the repeats that were identified by RepeatMasker, excluding small RNA, satellites, simple repeats and low complexity regions) composed, on average, 17.04% of a YAC sequence vs. 8.27% of a cosmid sequence. Contrary to what might be expected, low complexity repeats did not show a significant difference (0.31% vs. 0.3%).

**Table 1:** RepeatMasker results for YACs, cosmids, and fosmids (average per clone for each clone type).

	YACs			Cosmids			Fosmids		
Number of clones	515			2,520			107		
Total coverage (bp)	22,327,861			77,668,781			1,885,889		
Approximate portion of genome covered	22%			77%			2%		
Bases masked	21.42%			10.14%			12.14%		

	**Number of elements**	**Length occupied (bp)**	**Percentage of sequence (%)**	**Number of elements**	**Length occupied (bp)**	**Percentage of sequence (%)**	**Number of elements**	**Length occupied (bp)**	**Percentage of sequence (%)**

Retroelements	0.65	370.6	0.94	0.47	276.12	1.01	0.27	159.37	1.71
DNA transposons	22.48	4982.2	11.44	8.73	1743.45	5.69	5.75	1252.3	6.43
Unclassified	9.31	2306.1	4.65	2.57	510.22	1.58	1.99	469.55	1.97
* Total interspersed repeats*	32.44	7659	17.04	11.77	2529.79	8.27	8.01	1881.22	10.11
Small RNA	0.15	11.2	0.03	0.19	16.98	0.06	0.08	6.59	0.06
Satellites	3.11	942.26	2.52	0.93	156.76	0.51	0.75	143.05	0.74
Simple repeats	11.34	729.05	1.65	6.49	324.9	1.06	3.8	186.04	1.03
Low complexity	2.83	140.12	0.31	1.93	92.55	0.3	1.19	54.61	0.3

Comparison with the results that were obtained with the randomly generated clones showed that the differences between the clone types did not originate from their size. For all repeat types the percentage of sequence they covered in the “random YACs” was very similar to the percentages for “random cosmids” and “random fosmids,” unlike the results that were obtained with the real clones (Supplementary **Table S7**).

### THE RELATIVELY HIGH REPETITIVENESS OF THE SEQUENCES IN YACs PERSISTS EVEN WHEN CONTROLLING FOR THE CHROMOSOMAL LOCATION

**FIGURE 1:**
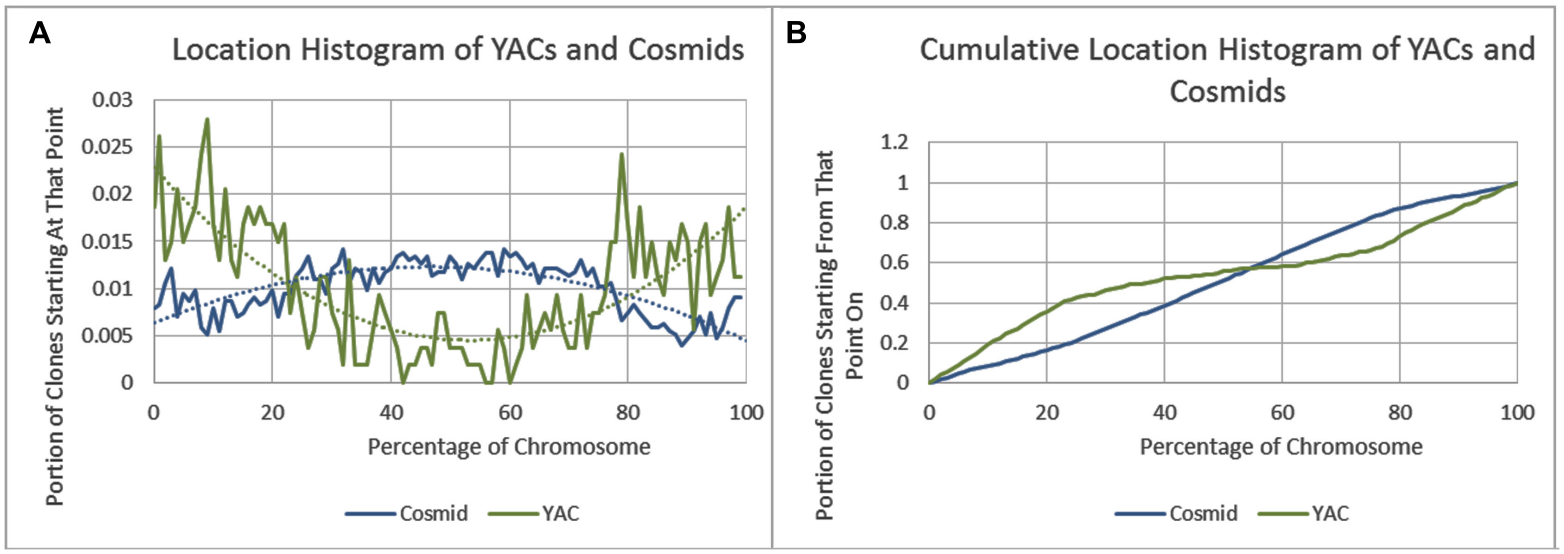
**Cosmid and YAC locations. (A)** The fraction of cosmids and YACs starting at each percent point of their chromosomes on average. **(B)** The cumulative probability distributions of cosmids and YACs on average. For each percentage point the fraction of clones starting at that point or further is given.

**FIGURE 2:**
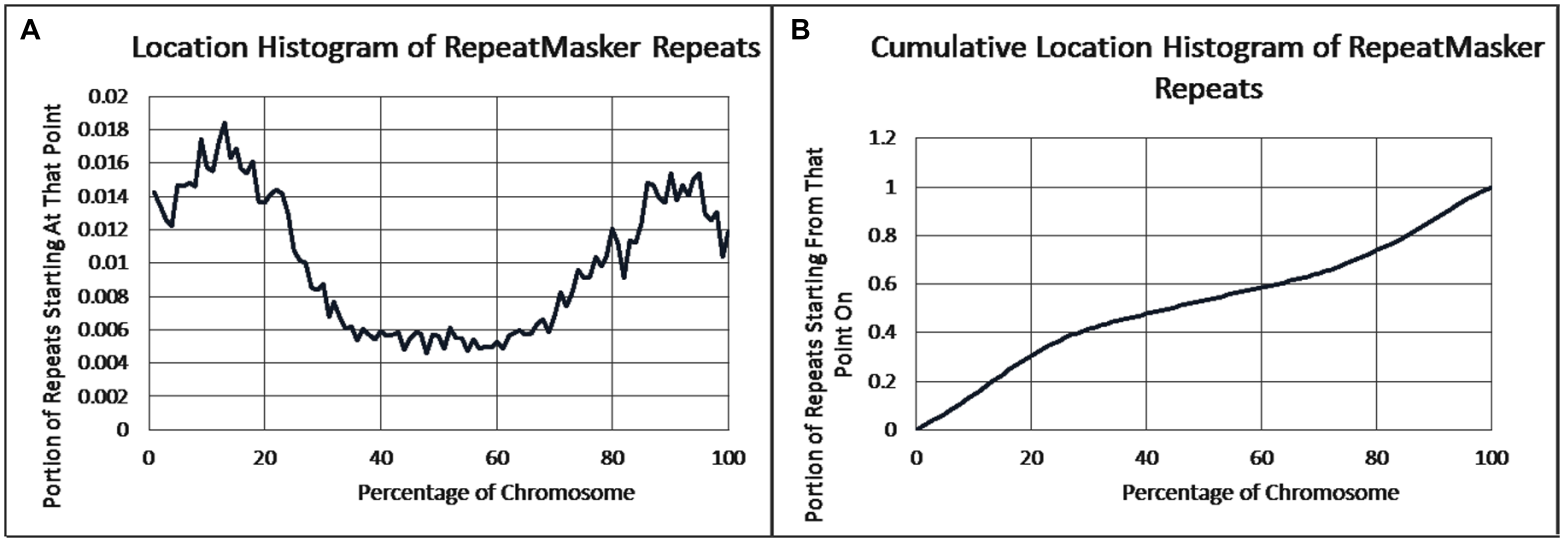
**Repeat locations. (A)** The fraction of repeats starting at each percent point of their chromosomes on average, as identified by RepeatMasker. **(B)** The cumulative probability distributions of repeats on average. For each percentage point the fraction of clones starting at that point or further is given.

**FIGURE 3:**
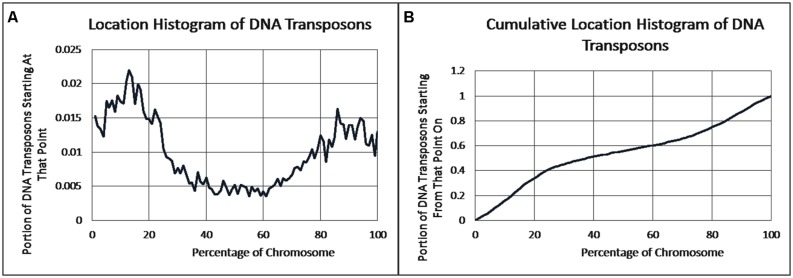
**DNA transposon locations. (A)** The fraction of DNA transposons starting at each percent point of their chromosomes on average, as identified by RepeatMasker. **(B)** The cumulative probability distributions of DNA transposons on average. For each percentage point the fraction of clones starting at that point or further is given.

As previously reported, we observed a significant difference in repetitiveness and transposon-frequency between the different chromosome regions (both *p* < 0.0001, *t*-test). The extreme quarters of chromosomes were more repetitive than the middle quarters of the chromosomes (bases masked – 18.69% vs. 6.99%), and were made up more of transposons (10.12% vs. 3.96%). We also found a noteworthy difference between chromosomal location distributions of the clone types. The vast majority of the YACs, a little over three quarters (396) were in the extreme quarters and the minority in the middle quarters (119), while the cosmids were mostly in the middle quarters (979 vs. 1,540 – a ratio of ∼2:3). We then ran RepeatMasker while taking into account this difference – comparing the differentiated groups between YACs and cosmids and within each clone type. Histograms of the locations of YACs and cosmids and the locations of repeats of any type are shown in **Figure 1**–**Figure 2**. A similar histogram for transposons, portraying the deviation more finely can be found in Figure **Figure 3**. To test the effect of the chromosomal location, we reran RepeatMasker separately on the extreme and on the middle quarters. In both cases the conclusions that were drawn from the previous analysis persisted. YAC-contained sequences were more repetitive, and were composed more of transposons than cosmids, although clones in the first and last quarters were overall more repetitive than their second and third quarter counterparts (Bases masked: YACs 22.83 and 16.71%, cosmids 15.69 and 6.62%, composed of transposons: YACs 12.78 and 7.01%, cosmids 8.49 and 3.91%).

### DIFFERENCES IN THE LENGTH OF YACs AND COSMIDS DO NOT ACCOUNT FOR THEIR REPETITIVENESS DIFFERENCE

There were no outstanding differences in repeat frequencies between the clones from the same type above and below the median length. Hence, the differences in repeat frequency between the clone types could not be explained by length variation.

### 18 YACs HAD NO REPETITIVE ELEMENTS

We examined the 18 YACs for which RepeatMasker did not find any repetitive elements. Out of these YACs, after removing overlaps with cosmids and fosmids, 12 were found to be smaller than 1 kb in length, 3 between 1 and 1.5 kb, and only 3 over 1.5 kb in length – Y68G5A (3.4 kb, after removing a 0.2 kb overlap; no genes in the non-overlapping portion), Y2C2A (8.5 kb, after removing an 8.5 kb overlap; part of the gene tag-80 in the non-overlapping portion) and Y53C12C (23.4 kb, after removing a 0.2 kb overlap; the gene *eyg-1* is enclosed in the non-overlapping portion).

### YACs ARE SIGNIFICANTLY ENRICHED FOR 21U piRNAs

An outstanding difference between the densities of protein-coding genes in YACs and cosmids was not found (a slightly higher density was found in cosmids: 182 genes per MB in YACs vs. 238 genes per MB in cosmids). tRNAs and rRNA are overrepresented in YACs – 94 YAC tRNA genes vs. 114 cosmid tRNA genes (4.2 vs. 1.5 genes per MB), and 10 YAC rRNA genes vs. 12 cosmid rRNA genes (0.45 vs. 0.15 genes per MB). Results for the CDS and ncRNA genes can be found in **Table 2**.

**Table 2:** Number and length statistics of protein-coding genes and ncRNAs in cosmids, fosmids and exclusive to YACs.

Clone and gene type	Number	Average length	Median length	LengthSD
Cosmid genes	18,071	1,119	948	1,090
Cosmid ncRNAs	11,093	102	83	138
Fosmid genes	325	1,193	999	1,143
Fosmid ncRNAs	817	43	21	59
YAC genes	3,882	1,058	894	913
YAC ncRNAs	10,728	31	21	58

The most striking result of our analysis was that ncRNAs of the type 21U piRNAs were dramatically overrepresented in YACs. A roughly similar absolute amount of ncRNA was found in YACs and cosmids despite the fact that YACs take up about a quarter of the length that cosmids take in the genome (10,799 vs. 11,108 ncRNA genes, respectively – corresponding with densities of 484 vs. 143 ncRNAs/MB). Further investigation revealed that the vast majority of the YAC ncRNAs are PIWI-interacting small RNAs, or 21U-RNAs (about 91%), while they are less than half of the cosmid ncRNAs (about 42.4%). Moreover, in terms of absolute quantities, YACs include more than twice as many 21U-RNAs as cosmids – 4,713 vs. 9,879 flip order to maintain consistency.

The majority of the DNA sequences that give rise to 21U piRNAs reside in two regions in chromosome IV (and also in another small region on chromosome IV) that are called “piRNA clusters” (Ruby et al., 2006). We analyzed these regions for repetitiveness and they turned out to be overall less repetitive compared to other regions, both on chromosome IV and on other chromosomes (the two major piRNA clusters had 8.15 and 11.51% of the bases masked as repetitive and the third cluster had 8.11% of the bases masked, compared to 11.89% for chromosome IV as a whole and 14.9% for chromosome I for example). Since the regions that encode for abundant YAC-specific piRNAs are relatively not repetitive, it raises the possibility that they are unclonable in *E. coli* for other reasons, perhaps due to toxicity (see Discussion). The longest four YACs, and seven of the longest ten YACs, were on chromosome IV (see **Table 3**). The longest four YACs alone hold 3,754 ncRNAs (roughly 35% of all ncRNA genes found in YACs), with the 6th longest holding 1,083 more. The longest 4 YACs also mapped specifically to one of the two piRNA clusters on chromosome IV.

**Table 3:** Longest 15 YACs, their locations, and lengths.

YAC Segment	Chromosome	Start position	End position	Length (kb)
Y73F8A	IV	15,226,338	15,549,111	323
Y105C5B	IV	15,863,724	16,185,028	321
Y105C5A	IV	15,548,990	15,863,827	315
Y57G11C	IV	14,637,373	14,950,845	313
Y75B8A	III	12,069,105	12,367,511	298
Y54G2A	IV	2,750,222	3,036,901	287
Y105E8A	I	14,333,265	14,610,766	278
Y51H4A	IV	16,471,836	16,741,455	270
Y116A8C	IV	16,900,192	17,160,330	260
Y111B2A	III	12,492,890	12,750,488	258
Y39B6A	V	18,958,057	19,204,189	246
Y53F4B	II	14,951,249	15,178,754	228
Y73B6BL	IV	6,286,329	6,502,271	216
Y47D3A	III	11,136,151	11,335,864	200
Y46G5A	II	12,680,715	12,878,522	198

While chromosome IV contains the vast majority of the YAC ncRNAs (93.3% vs. 44.1% of cosmid ncRNAs), it is not over-represented for YACs overall (21.8% of the YAC exclusive bases are on chromosome IV, which constitutes 17.4% of the nematode genome). When removing chromosome IV from the analysis, the discrepancy between YACs and cosmids on ncRNA content was flipped: instead of the ncRNAs being divided roughly equally between YACs and cosmids in terms of absolute numbers, when excluding chromosome IV, there were about 8.8 as many ncRNAs in cosmids as in YACs (6,182 vs. 702).

## DISCUSSION

We used a bioinformatic approach to probe DNA sequences that could be cloned in a eukaryote (yeast), but not in a prokaryote (bacteria). We found several major characteristics in such sequences, including high repetitiveness and enrichment for DNA transposons and 21U piRNAs. If these characteristics are typical to sequences of other organisms, it could in theory shed light on the barriers to genetic information transfer that exist between prokaryotes and eukaryotes, and should be interesting to validate experimentally.

The inability to efficiently clone repetitive sequences in bacteria is perhaps not biologically relevant, as it could simply stem from technical considerations. On the other hand, and while our results do not prove it in any way, it is possible that the inability to clone DNA transposons or piRNAs in bacteria has physiological importance.

Acquisition of vectors (cosmids or fosmids in this case) that carry mobile elements could be detrimental, if such genomic parasites are allowed to colonize or disrupt the genome of the host. It would be interesting to examine whether the type of transposons that we found to be enriched in YACs “jump” in *E. coli*, but not in *S. cerevisiae*, and if such transposition, should it be shown to occur, compromises the bacteria’s viability. Transposons that are not dependent on host factors could in theory transpose and confer damage, even upon lateral gene transfer.

piRNA-mediated RNA interference has a role in genome surveillance against foreign sequences in multiple organisms across the tree of life (animals and protists). One fascinating scenario that could be envisioned based on our data is that piRNAs might act *in trans*, to eliminate bacteria, *E. coli* in this case, with which *C. elegans* intimately interacts. Although *C. elegans* worms are not natural hosts of *E. coli*, the two organisms have been grown together in the lab for half a century. *C. elegans* and *E. coli* could in theory adapt to interact by these mechanisms, as piRNAs and piRNA pathway genes evolve rapidly, due to an “arms race” with transposons (Yi et al., 2014), and since piRNAs target rapidly evolving genes (Weick et al., 2014). The promoters of most piRNAs, which are autonomous transcriptional units in *C. elegans*, contain a consensus motif for binding of transcription factors (Weick et al., 2014). Thus in theory, possession of just one appropriate transcription factor could allow a host (such as *E. coli*) to transcribe most piRNAs. In flies, piRNA clusters act as “traps” in which transposons land (Malone and Hannon, 2009). It is not understood why most piRNAs in *C. elegans* are clustered together on chromosome IV. Perhaps the inability to transfer a large cluster of piRNAs from *C. elegans* to *E. coli* is one of the reasons.

Characterization of DNA sequences that are lethal to bacteria but not to yeast could be applicative (Sorek et al., 2007; Kimelman et al., 2012). Given further research and validation, it could be possible in the future to combine in gene therapy, for example, piRNA-encoding DNA sequences, and thus to affect bacteria, while sparing the eukaryote hosts with which they interact.

## Conflict of Interest Statement

The Guest Associate Editor Eva Jablonka declares that, despite being affiliated to the same institution as the authors, the review process was handled objectively and no conflict of interest exists. The authors declare that the research was conducted in the absence of any commercial or financial relationships that could be construed as a potential conflict of interest.

## SUPPLEMENTARY MATERIAL

Table S1 **RepeatMasker results for the various groups of clones, averaged per clone for each clone type**.

Table S2 **RepeatMasker results for the various groups of clones, median per clone for each clone type**.

Table S3 **RepeatMasker results for the various groups of clones, summed per clone for each clone type**.

Table S4 **RepeatMasker results for the various groups of clones, number of clones for each clone type that are non-zero**.

Table S5 **RepeatMasker results for the various groups of clones, the minimal result per clone for each clone type and the clones with that value**.

Table S6 **RepeatMasker results for the various groups of clones, the maximal result per clone for each clone type and the clones with that value**.

Table S7 **RepeatMasker results for the randomly-selected genomic segments with the same size distributions asYACs, cosmids, and fosmids (averaged per segment for each clone type)**.
